# plantMASST - Community-driven chemotaxonomic digitization of plants

**DOI:** 10.1101/2024.05.13.593988

**Published:** 2024-05-14

**Authors:** Paulo Wender P. Gomes, Helena Mannochio-Russo, Robin Schmid, Simone Zuffa, Tito Damiani, Luis-Manuel Quiros-Guerrero, Andrés Mauricio Caraballo-Rodríguez, Haoqi Nina Zhao, Heejung Yang, Shipei Xing, Vincent Charron-Lamoureux, Desnor N. Chigumba, Brian E. Sedio, Jonathan A. Myers, Pierre-Marie Allard, Thomas V. Harwood, Giselle Tamayo-Castillo, Kyo Bin Kang, Emmanuel Defossez, Hector H. F. Koolen, Milton Nascimento da Silva, Consuelo Yumiko Yoshioka e Silva, Sergio Rasmann, Tom W. N. Walker, Gaëtan Glauser, José Miguel Chaves-Fallas, Bruno David, Hyunwoo Kim, Kyu Hyeong Lee, Myeong Ji Kim, Won Jun Choi, Young-Sam Keum, Emilly J. S. P. de Lima, Lívia Soman de Medeiros, Giovana A. Bataglion, Emmanoel V. Costa, Felipe M. A. da Silva, Alice Rhelly V. Carvalho, José Diogo E. Reis, Sônia Pamplona, Eunah Jeong, Kyungha Lee, Geum Jin Kim, Yun-Seo Kil, Joo-Won Nam, Hyukjae Choi, Yoo Kyong Han, Si Young Park, Ki Yong Lee, Changling Hu, Yilun Dong, Shengmin Sang, Colin R. Morrison, Ricardo Moreira Borges, Andrew Magno Teixeira, Seo Yoon Lee, Bum Soo Lee, Se Yun Jeong, Ki Hyun Kim, Adriano Rutz, Arnaud Gaudry, Edouard Bruelhart, Iris F. Kappers, Rumyana Karlova, Mara Meisenburg, Roland Berdaguer, J. Sebastián Tello, David Henderson, Leslie Cayola, S. Joseph Wright, David N. Allen, Kristina J. Anderson-Teixeira, Jennifer L. Baltzer, James A. Lutz, Sean M. McMahon, Geoffrey G. Parker, John D. Parker, Trent R. Northen, Benjamin P. Bowen, Tomáš Pluskal, Justin J. J. van der Hooft, Jeremy J. Carver, Nuno Bandeira, Benjamin S. Pullman, Jean-Luc Wolfender, Roland D. Kersten, Mingxun Wang, Pieter C. Dorrestein

**Affiliations:** 1 Collaborative Mass Spectrometry Innovation Center, Skaggs School of Pharmacy and Pharmaceutical Sciences, University of California, San Diego, La Jolla, CA, USA; 2 Skaggs School of Pharmacy and Pharmaceutical Sciences, University of California, San Diego, La Jolla, CA, USA; 3 Institute of Organic Chemistry and Biochemistry of the Czech Academy of Sciences, Prague, Czech Republic; 4 Institute of Pharmaceutical Sciences of Western Switzerland, University of Geneva, CMU, 1211 Geneva, Switzerland; 5 School of Pharmaceutical Sciences, University of Geneva, CMU, 1211 Geneva, Switzerland; 6 College of Pharmacy, Kangwon National University, Chuncheon, Republic of Korea; 7 Department of Medicinal Chemistry, University of Michigan, Ann Arbor, MI, USA; 8 Program in Chemical Biology, University of Michigan, Ann Arbor, MI, USA; 9 Department of Integrative Biology, University of Texas at Austin, Austin, TX, USA; 10 Smithsonian Tropical Research Institute, Republic of Panama; 11 Department of Biology, Washington University in St. Louis, St. Louis, MO, USA; 12 Department of Biology, University of Fribourg, 1700 Fribourg, Switzerland; 13 The DOE Joint Genome Institute, Lawrence Berkeley National Laboratory, One Cyclotron Road, Berkeley, CA, 94720, United States; 14 Centro de Investigaciones en Productos Naturales (CIPRONA), Universidad de Costa Rica, San José 11501–2060, Costa Rica; 15 Escuela de Química, Universidad de Costa Rica, 2061 San José, Costa Rica; 16 College of Pharmacy and Research Institute of Pharmaceutical Sciences, Sookmyung Women’s University, Seoul, Republic of Korea; 17 Institute of Biology, University of Neuchâtel, 2000 Neuchâtel, Switzerland; 18 Botanical garden of Neuchâtel; 19 University of the State of Amazonas, Manaus, Brazil; 20 Laboratory of Liquid Chromatography, Federal University of Pará, Belém 66075-110, Brazil; 21 Institute of Exact and Natural Sciences, Federal University of Pará, Belém 66075-110, Brazil; 22 Institute of Health Sciences, Faculty of Pharmacy, Federal University of Pará, Belém 66075-110, Brazil; 23 Neuchâtel Platform of Analytical Chemistry, University of Neuchâtel, 2000 Neuchâtel, Switzerland; 24 Department of Biology and Whitney R. Harris World Ecology Center, University of Missouri-St. Louis, St. Louis, MO 63121, USA; 25 Green Mission Pierre Fabre, Institut de Recherche Pierre Fabre, 3 Avenue Hubert Curien, BP 13562, 31562 Toulouse, France; 26 College of Pharmacy and Integrated Research Institute for Drug Development, Dongguk University, 32, Dongguk-ro, Goyang, Gyeonggi-do 10326, Korea; 27 Federal University of São Paulo, Diadema, Brazil; 28 Department of Chemistry, Federal University of São Paulo, Diadema, SP, 09972-270, Brazil; 29 Federal University of Amazonas, Manaus, Brazil; 30 Department of Pharmacology, College of Medicine, Dongguk University, Gyeongju, Gyeongsangbuk-do, Republic of Korea; 31 Research Institute of Cell Culture, Yeungnam University, Gyeongsan, Republic of Korea; 32 College of Pharmacy, Yeungnam University, Gyeongsan, Republic of Korea; 33 College of Pharmacy and Inje Institute of Pharmaceutical Sciences and Research, Inje University, Gimhae, Gyeongnam 50834, Republic of Korea; 34 Core Research Support Center for Natural Products and Medical Materials, Yeungnam University, Gyeongsan, Republic of Korea; 35 College of Pharmacy, Korea University, Sejong, Republic of Korea; 36 Laboratory for Functional Foods and Human Health, Center for Excellence in Post-Harvest Technologies, North Carolina Agricultural and Technical State University, North Carolina Research Campus, Kannapolis, NC, USA; 37 Brackenridge Field Laboratory, University of Texas at Austin, Austin, TX, USA; 38 Walter Mors Institute of Research on Natural Products, Federal University of Rio de Janeiro, Rio de Janeiro, Brazil; 39 School of Pharmacy, Sungkyunkwan University, Suwon 16419, Republic of Korea; 40 Institute of Molecular Systems Biology, ETH Zurich, Zurich, Switzerland; 41 Laboratory of Plant Physiology, Plant Sciences Group, Wageningen University, Wageningen, The Netherlands; 42 Latin America Department, Missouri Botanical Garden, St. Louis, MO, USA,; 43 Missouri Botanical Garden, St. Louis, MO, USA; 44 Herbario Nacional de Bolivia, Universidad Mayor de San Andrés, La Paz, Bolivia; 45 Smithsonian Tropical Research Institute, Balboa, Republic of Panama; 46 Department of Biology, Middlebury College, Middlebury, VT, USA; 47 Conservation Ecology Center, Smithsonian’s National Zoo & Conservation Biology Institute, Front Royal, VA, USA; 48 Department of Biology, Wilfrid Laurier University, Waterloo, ON, Canada; 49 Department of Wildland Resources, Utah State University, Logan, UT, USA; 50 Smithsonian Environmental Research Center, Edgewater, MD, USA; 51 Forest Global Earth Observatory, Smithsonian Tropical Research Institute, Panama City, Panama; 52 Forest Ecology Group, Smithsonian Environmental Research Center, Edgewater, MD, USA; 53 Environmental Genomics and Systems Biology Division and the DOE Joint Genome Institute, Lawrence Berkeley National Laboratory, Berkeley, CA, 94720, United States; 54 Bioinformatics Group, Wageningen University & Research, 6708 PB Wageningen, the Netherlands; 55 Department of Biochemistry, University of Johannesburg, Johannesburg 2006, South Africa; 56 Center for Computational Mass Spectrometry, Department of Computer Science and Engineering, Skaggs School of Pharmacy and Pharmaceutical Sciences, University of California San Diego, San Diego 92093-0404, United States; 57 Department of Computer Science, University of California Riverside, Riverside, CA, 92521, United States

## Abstract

Understanding the distribution of hundreds of thousands of plant metabolites across the plant kingdom presents a challenge. To address this, we curated publicly available LC-MS/MS data from 19,075 plant extracts and developed the plantMASST reference database encompassing 246 botanical families, 1,469 genera, and 2,793 species. This taxonomically focused database facilitates the exploration of plant-derived molecules using tandem mass spectrometry (MS/MS) spectra. This tool will aid in drug discovery, biosynthesis, (chemo)taxonomy, and the evolutionary ecology of herbivore interactions.

Earth harbors around 450 plant families, 16,000 genera, and 350,000 vascular plant species^1^. Plants are essential to our planet’s health through their role in converting CO_2_ into oxygen and energy, sustaining animal life, including our own, and have been used by humans to treat diseases. Despite their ecological, nutritional, and medicinal value, the taxonomic distribution of plant metabolites is hard to establish. This is due to the absence of a common open-access spectral MS plant database and search engines. Furthermore, not every species distributed in the plant kingdom has been studied and not every imaginable metabolite has been discovered. Most chemotaxonomic studies of plants are limited to specific clades of the taxonomic tree^2–5^ and rely on data availability of known compounds in structural and spectral databases^6–14^, limiting the search of yet-to-be-characterized molecules. However, creating a chemophenetic plant metabolite inventory of all plant species found worldwide is within reach as mass spectrometry (MS) technologies and algorithms are continuously improving. To facilitate this, we have created plantMASST, a taxonomically-informed mass spectrometry search tool for plant metabolites within the Global Natural Products Social Molecular Networking (GNPS) ecosystem. plantMASST builds on the the approach taken for microbeMASST^15^, creating a digital inventory of untargeted plant metabolomics data. This enables the querying of MS/MS spectra corresponding to known and unknown molecules within a curated database of LC-MS/MS data from plant extracts, with results mapped across the plant taxonomic tree. As of May 2024, plantMASST curated reference datasets containing LC-MS/MS data from 19,075 plant extracts with over 100 million MS/MS spectra linked to their respective taxonomical information (Figure 1a). The plantMASST reference database results from community contributions and metadata curation from 90 scientists worldwide and it now includes 246 botanical families, 1,469 genera, and 2,793 species. To increase plantMASST coverage, we encourage the community to deposit new plant datasets in MassIVE (https://massive.ucsd.edu/) with associated metadata in the ReDU template^16^.

A single MS/MS spectrum can be searched in plantMASST through its web interface (https://masst.gnps2.org/plantmasst/). The search output is a list of all data files where the queried MS/MS spectrum (within the user-defined scoring criteria) has been observed. Additionally, an interactive taxonomic tree of the results is generated, which can be easily explored (Figure 1b,c). The search is accomplished by providing either a Universal Spectrum Identifier (USI)^17^ or a precursor *m/z* and a corresponding list of fragment ions as *m/z*-intensity-pairs (Supplementary Figure S1a). Although the parameters are adjustable, the web interface provides the following default values: precursor and fragments mass tolerance set at 0.05 Da and cosine similarity of 0.7 with at least three matched fragment ions shared between the queried spectrum and the spectra available in plantMASST. “Analog search” can also be enabled by the user to explore MS/MS spectra from putative-related structural analogs or different ion forms of the same molecules (*e.g.*, different adducts, multimers, and in-source fragment ions). Moreover, plantMASST automatically performs spectral library searches against the reference libraries publicly available within the GNPS environment for direct metabolite annotation through MS/MS spectral matching. Building on the concepts of MASST^18^ and fastMASST^19^, plantMASST returns results within seconds and includes the taxonomic distribution of metabolites at once. The interactive taxonomic tree (and links to related tools in the GNPS ecosystem) used to visualize the results was built using NCBI^20^ taxonomy (Supplementary Figure S1b). Finally, users can explore matches inspecting the MS/MS mirror plots, with highlighted shared peaks, using the Metabolomics Spectrum Resolver^21^ and access the corresponding LC-MS/MS files using the GNPS Dashboard^22^.

Here, we highlight the use of plantMASST through three representative applications on natural product discovery and one about how to explore public human diet intervention datasets. First, researchers may desire to explore the taxonomic distribution of known plant-derived molecules. Therefore, as proof of concept, we investigated moroidin. This bicyclic octapeptide belongs to the BURP-domain-derived ribosomally synthesized and post-translationally modified peptides (RIPPs) family^23^, which was first isolated from *Dendrocnide moroides* Wedd. (Urticaceae)^24^ and *Celosia argentea* L. (Amaranthaceae)^25^. This molecule was described to induce apoptosis in the A549 human non-small cell lung cancer cell line and it has been gaining attention in the search for new drugs for cancer treatment^26^. We used plantMASST to search an MS/MS spectrum of the [M + 2H]^2+^ ion of moroidin and found four potential producer species across two different plant families, including yellow bauhinia (*Bauhinia tomentosa* Vell., Fabaceae) which was not a known producer of moroidin. Moroidin identification in the extract of *B. tomentosa* seeds was confirmed as Metabolite Identification level 1 by comparison to an authentic standard (Figure 1b) according to the Metabolomics Standard Initiative (MSI, Supplementary Figure S4)^27^. Another example of natural product discovery across the taxonomic domain is showcased by searching for piperlongumine, an amide alkaloid originally isolated from the *Piper* genus (Piperaceae), which has a reported antitumor activity^28^ (Figure 1c). This molecule is also currently being investigated for the treatment of glioblastoma^29^. Utilizing plantMASST, we observed that the piperlongumine MS/MS spectrum was also detected in two other botanical species: *Gymnotheca chinensis* Decne. (Saururaceae) and *Clematis apiifolia* DC. (Ranunculaceae), which has not been previously reported. MS/MS and retention time matching with a commercial standard allowed us to confirm the presence of piperlongumine in both species and achieve level 1 identification (Figure 1c)^27^. This expands the known natural reservoirs of piperlongumine beyond its primary source, *Piper longum* L. (Piperaceae), and highlights the versatility and efficacy of plantMASST in discovering pharmaceutically relevant phytochemicals in diverse plant families. It also suggests new research questions, such as why the production of these alkaloids is observed in such diverse plant species. Other plantMASST representative applications, as such caffeine, reserpine, icaridin, lutein, methoxsalen, and tryptophan, can be found in Supplementary Figures S1, S3, S4, and S6.

Beyond demonstrating the potential of plantMASST for highlighting specific plant metabolites with a narrow distribution within the plant kingdom, we built on recent observations that human neuroactives are also present in plants^30,31^. Many of them are involved in the signaling communication and adaptation of plants^31^. However, it is yet unknown to what extent they are distributed across the plant kingdom. As no report exists at the moment, we used plantMASST to search the MS/MS spectra of acetylcholine, dopamine, serotonin, glutamate, gamma-aminobutyric acid (GABA), and norepinephrine, all known neuroactives also produced by humans. Additionally, we searched for cannabidiol (CBD) and tetrahydrocannabinol (THC), two plant-derived metabolites known to affect human brain physiology for comparison (Figure 2a,b). Serotonin, also known as 5-HT, was detected in 61 out of 246 plant families available in plantMASST, with a notable prevalence in species belonging to the Malpighiaceae family, including *Banisteriopsis* genus (Figure 2b). This observation suggests this genus is a potential source of neuroactives such as serotonin. Additionally, it may also explain the neuroactive properties of species of this genus, such as *Banisteriopsis caapi* C.V. Morton (Malpighiaceae), which is used as the main ingredient of Ayahuasca, an indigenous beverage traditionally used in the Northwestern Amazon to treat mental health disorders^32^. These plants could be further evaluated for serotonin-mediated effects on breathing, sleep, arousal, and seizure control^33,34^. Other neuroactives were found in specific plant families. For instance, tryptamine was detected only in *Solanum lycopersicum* L. (Solanaceae); CBD and THC, two molecules that have neurological effects on humans, were found only in *Cannabis sativa* (Cannabaceae)*.* Therefore, plantMASST can also enable users to explore the diversity of neuroactives in plants and highlight plants that could be further investigated for potential pharmacological applications or the extraction of bioactive compounds with medicinal properties.

Although plantMASST and the underlying reference database can be leveraged in many ways, we showcase a last example of how plantMASST can be used to detect human dietary consumption of plants. We reanalyzed all the MS/MS spectra from two public metabolomics datasets of human fecal data of diet-related studies; one comparing vegan *vs* omnivore diets^35,36^, and the other American *vs* Mediterranean diets^37^. We observed a higher percentage of MS/MS matches to plant metabolites in the vegan group when compared to the omnivore group (Figure 2c). Interestingly, also the subjects consuming the Mediterranean diet had more MS/MS matches to plant metabolites compared to the American diet (Figure 2d). These results suggest that plantMASST could also be used to define the nature of plant-derived dietary patterns.

It is important to bear in mind certain limitations when interpreting the results of plantMASST since the current reference database contains diverse experimental and acquisition conditions. This includes the plant organ, plant growth conditions, the biome of origin, extraction methods, sample preparation, instrument geometries, and defined collision energies, among others. Therefore, even if the plant is known to be a producer of a compound of interest, a plantMASST match might be missed because of fewer fragment ion matches to low-intensity MS/MS ions or because no MS/MS of the compound was triggered due to low precursor ion intensity. If the users decrease the tolerances of minimum MS/MS fragment peaks to find more matches, it may result in more false positive matches. Further, although many MS/MS spectra will be quite specific to one molecule (*e.g.*, moroidin and reserpine, Supplementary Figure S2a), isomers that have the same mass, can have nearly identical MS/MS spectrum, which is the case of quercetin and morin (Supplementary Figure S2b). This means that in some cases the taxonomic interpretation of a family of molecules may not map well to the individual molecules. Finally, there are currently only 2,793 species of plants, representing a fraction of the species that exist. Despite these challenges, plantMASST represents an important advance in our ability to map the plant taxonomic distribution of metabolites, especially as researchers continue to expand the taxonomic curation of untargeted plant metabolomics data. We expect that plantMASST will potentially have profound implications for the fields of drug discovery, nutrition, and chemical ecology.

## Methods

### Data collection and curation

To enable the taxonomic search of known and unknown compounds and to make a dent in capturing chemotaxonomic data from all available plant extracts, 211 publicly available MS/MS datasets in the GNPS/MassIVE were manually compiled and each file, within these datasets, was taxonomically defined possibly to species level. For the samples in which the species was not known, the closest known taxonomic rank was defined (e.g., genus or family). These datasets consisted of 20,209 unique LC-MS/MS files, representing plant extracts inclusive of all plant tissue types (leaves, stems, seeds, among others) and habits (trees, shrubs, lianas, herbs, etc). To collect this number of samples, we made an open call to the scientific community to deposit plant-related datasets in GNPS/MassIVE, which led to the deposition of an additional 25 datasets between December 2022 and March 2023, resulting from the efforts of 12 research groups across the world. All the collected information of each file part of plantMASST is available on GitHub and contains the following information: the path of each file, the filename in the format ‘Dataset/Filename’, the MassIVE ID, the taxon name, the NCBI Taxonomy ID, ReDU availability, whether the file is relative to a blank or a QC, and the USI of the file.

To get the NCBI taxonomy IDs, the datasets containing ReDU metadata were matched to NCBI IDs. When only a table containing the sample names was provided, the NCBI taxonomy IDs were manually retrieved from the NCBI Taxonomy web browser (https://www.ncbi.nlm.nih.gov/taxonomy).

### plantMASST taxonomic tree generation

The plantMASST taxonomy tree was created with R Studio 4.2.2 and Python 3.10. Only distinct NCBI IDs (n = 3,173) were kept in the database. To obtain the complete lineage of every NCBI ID^38^, the ‘taxize’ package (v.0.9.100) was used, namely its categorization function. The main taxonomic ranks (kingdom to species) along with subgenus, subspecies, and variations were maintained to create taxonomic trees with an equal number of taxa nodes. After importing the NCBI ID list for every taxon into Python, a taxonomic tree was created using the ETE toolkit^39^. After that, the created Newick tree was transformed into JSON format, and details like taxonomic rank and number of accessible samples were added. All taxonomic entries in plantMASST are classified according to the NCBI taxonomy^20^, which includes 246 families, 1,469 genera, and 2,793 species. Thus, while plantMASST encompasses more than 50% of the world’s botanical families present in the current community curation, less than 1% of species are represented. The representation of the plant data will continue to grow as the scientific community continues to contribute to expanding publicly available plant-related datasets.

### MASST searching

plantMASST can be used as a web application by accessing https://masst.gnps2.org/plantmasst/. This web application was built using Dash and Flask open-source libraries for Python (https://github.com/mwang87/GNPS_MASST/blob/master/dash_plantmasst.py). Searches in the web interface can be done by inputting a Spectrum USI or the spectrum peaks of an MS/MS spectrum and further clicking on ‘Search plantMASST by USI’ or ‘Search plantMASST by Spectrum Peaks’. The default search parameters are cosine threshold: 0.7; minimum matched peaks: 3; precursor mass and fragment tolerance: 0.05 Da; analog search: no. Users familiar with Python can also create batch searches via the command line, which produces results for plantMASST, microbeMASST, and foodMASST. Details can be found at https://zenodo.org/records/10909241. With this approach, users have to provide either a CSV or TSV file containing the USIs to be searched or an MGF file containing precursors and MS/MS spectra of the ions. The same settings described in the API web interface such as minimum cosine, *m/z* tolerance, precursor *m/z*, and minimum fragments matched, are adjustable. To create the resulting taxonomic tree, the JSON file of the complete plantMASST taxonomic tree is filtered and converted into a D3 JavaScript object that can be visualized as an HTML file.

### plantMASST applications

plantMASST can be used in a variety of scenarios. First, we showed the taxonomic distribution of specific metabolites present in the GNPS public libraries (moroidin, caffeine, reserpine, icaridin, lutein, methoxsalen, and piperlongumine). These searches were done using the web interface and the default parameters. Second, selected neuroactives with MS/MS spectra available in the GNPS public libraries (acetylcholine, dopamine, GABA, glutamate, norepinephrine, serotonin, CBD, THC, and tryptamine) were searched against plantMASST with the web interface using the default parameters. The matches obtained were manually inspected via mirror plots and the low-quality matches were filtered out. The tables containing the taxonomic distribution were downloaded and combined to visualize them using the Interactive Tree of Life (iTOL)^40^. A heatmap was generated in Python (version 3.11) to show the plantMASST matches of these neuroactives to different plant species. The packages ‘pandas’ (version 2.2.2) and ‘plotly’ (version 5.21.0) were used for this analysis. Finally, we re-analyzed two diet-related MS/MS studies (vegan vs omnivore diet: MSV000086989; American vs Mediterranean diet: MSV000093005). The raw data was processed in MZmine3 (version 3.4.27). The MZmine3 batch files used in each study are available on GitHub (https://github.com/helenamrusso/plantmasst), in addition to the generated output files. The generated .mgf files were used as input for the plantMASST batch search (https://github.com/robinschmid/microbe_masst) using the following parameters: cosine threshold: 0.7; minimum matched peaks: 4; precursor mass and fragment tolerance: 0.02 Da; analog search: off. Boxplots showing the percentages of matches to plantMASST in each sample were generated in Python using the ‘seaborn’ package (version 0.11.2). The Shapiro-Wilk test was used to assess the normality of the data.

### LC-MS/MS analysis

#### Retention time matching for moroidin

Moroidin was isolated and purified as described^23^ from *Celosia argentea* flower and seed material. All materials were purchased from Fisher Scientific unless otherwise noted. *Celosia argentea* seeds were purchased from Seedville^™^, and *Bauhinia tomentosa* seeds were purchased from rarepalmseeds.com^™^. 0.2 g of *C. argentea* seeds or *B. tomentosa* seeds were each ground with mortar and pestle, extracted with 3 mL methanol for 1 h shaking at 200 rpm at 37 °C in a 7 mL glass scintillation vial (Fisher Scientific 03–337-26). The methanol extracts were dried under nitrogen gas and resuspended in 3 mL of deionized water. The resuspensions were partitioned twice with hexane (1:1, v/v), partitioned twice with ethyl acetate (1:1, v/v), and extracted once with 3 mL n-butanol. The n-butanol extract was dried *in vacuo* in a Thermo Scientific SPD140P1 speed vac and resuspended in 2 ml of 80% methanol for LC-MS/MS analysis. The moroidin standard had a final concentration of 500 nM in 80% methanol.

LC-MS/MS analysis for moroidin analysis was carried out on a Thermo H-ESI-Q-Exactive Orbitrap mass spectrometer coupled to a Thermo Vanquish ultra-HPLC system. H-ESI parameters were as follows: spray voltage: +4.2 kV; ion transfer tube temperature: 320 °C; S-lens RF: 50 (arb units); sheath gas flow rate: 35 (arb units); Sweep Gas: 0, and auxiliary gas flow rate: 3 (arb. units). The LC-MS/MS settings were as follows: injection volume 5 μl; LC, Phenomenex Kinetex 2.6 μm C18 reverse phase 100 Å 150 ×3 mm LC column; LC gradient, solvent A, 0.1% formic acid; solvent B, acetonitrile (0.1% formic acid); 0 min, 10% B; 5 min, 60% B; 5.1 min, 95% B; 6 min, 95% B; 6.1 min, 10% B; 9.9 min, 10% B; 0.5 ml min^−1^; MS, positive ion mode; full MS, resolution 70,000; mass range 400–1,200 *m/z*; dd-MS^2^ (data-dependent MS/MS), resolution 17,500; AGC target 1 × 10^5^, loop count 5, isolation width 1 *m/z*, collision energy 25 eV and dynamic exclusion 0.5 s. LC-MS/MS data were analyzed with QualBrowser in the Thermo Xcalibur software package (v4.3.73.11).

#### Retention time matching for piperlongumine

Piperlongumine was obtained as a commercial standard (>97 % HPLC quality, SML0021, Sigma-Aldrich) to enable the confirmation of the presence of this compound in additional plant extracts. LCMS grade acetonitrile, water, and formic acid (all Optima^®^ LCMS Grade, Fisher Scientific, Germany) were used for the analyses. The Pierre Fabre Laboratories supplied the dried extracts for the *Gymnotheca chinensis* leaves (V113160) and green stems (V113161), *Clematis apiifolia* leaves (V113131), roots (V113132) and green stems (V113133). The extracts were prepared as described by Allard *et al.* (2023)^41^. All the samples were dissolved in methanol at a concentration of 5 mg/mL. The piperlongumine standard was prepared at a concentration of 100 μg/mL in methanol.

Analyses were performed with a Vanquish Horizon (Thermo Scientific) equipped with a binary pump H, a dual split sampler HT and a Diode Array detector FG coupled to an Orbitrap Exploris 120 mass spectrometer (Thermo Scientific), and a Corona Veo RS Charged Aerosol Detector (CAD, Thermo Scientific). The Orbitrap employs a heated electrospray ionization source (H-ESI) with the following parameters: spray voltage: +3.5 kV; ion transfer tube temperature: 320.00 °C; vaporizer temperature: 320.00 °C; S-lens RF: 45 (arb units); sheath gas flow rate: 35.00 (arb units); Sweep Gas (arb): 1, and auxiliary gas flow rate: 10.00 (arb. units).

The mass analyzer was calibrated using a mixture of caffeine, methionine-arginine-phenylalanine-alanine-acetate (MRFA), sodium dodecyl sulfate, sodium taurocholate, and Ultramark 1621 in an acetonitrile/methanol/water solution containing 1% formic acid by direct injection. Control of the instruments was done using Thermo Scientific Xcalibur software v. 4.6.67.17. Full scans were acquired at a resolution of 30,000 FWHM (at *m/z* 200) and MS^2^ scans at 15000 FWHM in the range of 100–1000 *m/z*, with 1 microscan, time (ms): 200 ms, an RF lens (%): 70; AGC target custom (Normalized AGC target (%): 300); maximum injection time (ms): 130; Microscans: 1; data type: profile; Usue EASY-IC(TM): ON. The Dynamic exclusion mode: Custom; Exclude after n times: 1; Exclusion duration (s): 5; Mass tolerance: ppm; low: 10, high: 10, Exclude isotopes: true. Appex detention: Desired Apex Window (%): 50. Isotope Exclusion: Assigned and unassigned with an exclusion window (*m/z*) for unassigned isotopes: 8. The Intensity threshold was set to 2.5E^5^ and a targeted mass exclusion list was used. The centroid data-dependent MS^2^ (dd-MS^2^) scan acquisition events were performed in discovery mode, triggered by Apex detection with a trigger detection (%) of 300 with a maximum injection time of 120 ms, performing 1 microscan. The top 3 abundant precursors (charge states 1 and 2) within an isolation window of 1.2 *m/z* were considered for MS/MS analysis. For precursor fragmentation in the HCD mode, a normalized collision energy of 15, 30, and 45% was used. Data was recorded in profile mode (Use EASY-IC(TM): ON).

The chromatographic separation was done on a Waters BEH C18 column (100 × 2.1 mm i.d., 1.7 μm, Waters, Milford, MA) using a gradient as follows (time (min), %B):0.00, 2; 3.1, 2; 17.36, 99; 23.12, 99; 23.60, 2; 26.00, 2. The mobile phases were (A) water with 0.1% formic acid and (B) acetonitrile with 0.1% formic acid. The flow rate was set to 500 μL/min, the injection volume was 3 μL, and the column was kept at 40 °C. The PDA detector was used from 210 to 400 nm with a resolution of 1.2 nm. The CAD was kept at 40 °C, 5 bar N_2_, and power function 1 for a data collection rate of 20 Hz.

## Supplementary Material

Supplement 1

## Figures and Tables

**Figure 1.**
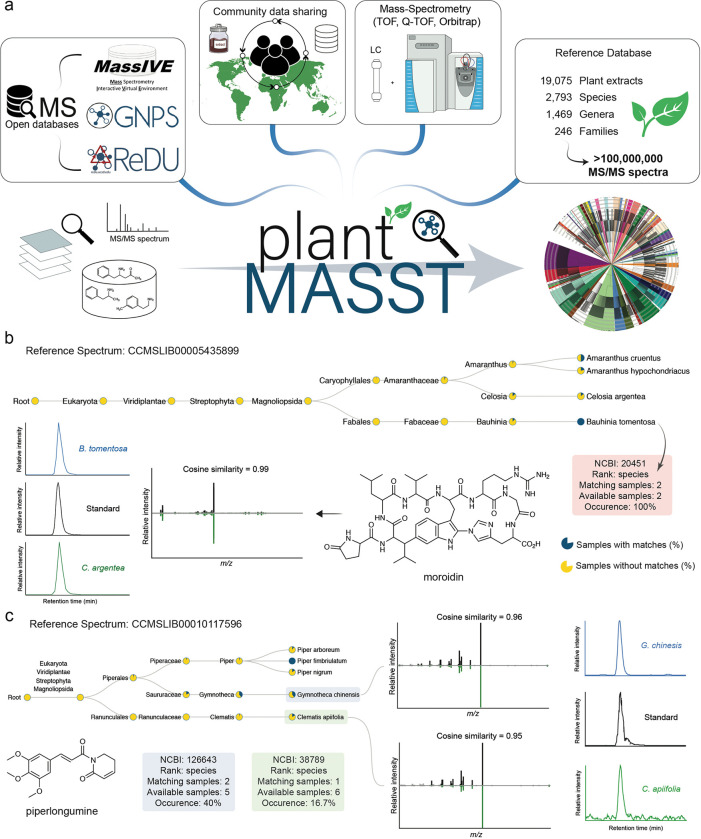
Schematic overview of plantMASST infrastructure and output. a) The creation of the plantMASST reference database involved utilizing 19,075 community-curated LC-MS/MS data and knowledge from MassIVE, GNPS^12^, and ReDU^16^. b) Example of plantMASST output interpretation of a specialized metabolite known to be produced by specific plants, moroidin (the molecule was confirmed using standard, Supplementary Figure S4). Pie charts display the percentage of matches within that taxonomic level detected against the plantMASST database. Blue indicates the percentage of samples with matches and yellow without matches. The reference MS/MS spectrum for moroidin (CCMSLIB00005435899) is available in the GNPS library. c) Output of piperlongumine (CCMSLIB00010117596) search. Mainly produced by *Piper* species, the molecule was also confirmed, for the first time, to be present in *Gymnotheca chinensis* and *Clematis apiifolia* leaf extracts via MS/MS and retention time matching to its commercial standard. In the mirror plots, the spectrum on the top is relative to piperlongumine, and the one on the bottom is a match to plantMASST. More tissues were also matched and the chromatograms can be found in Supplementary Figure S5.

**Figure 2.**
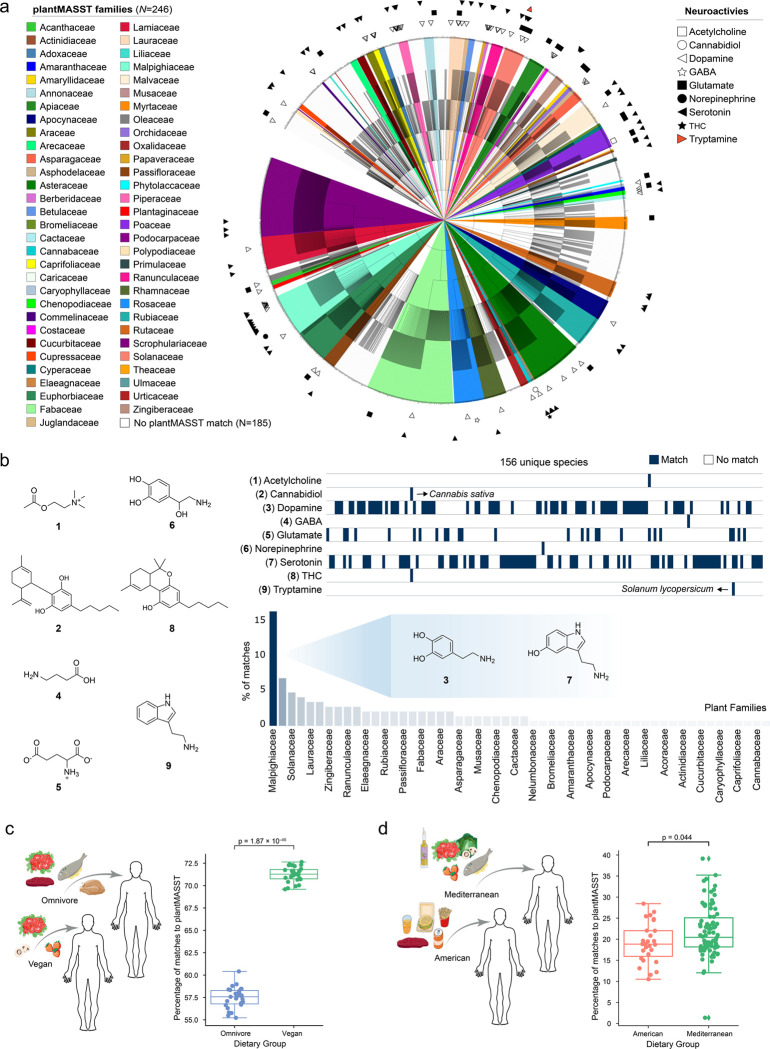
Use of plantMASST in the search for neuroactives in plants and plant-derived small molecules in public data sets of fecal samples of humans. a) Taxonomic distribution of neuroactive compounds across plant families. b) Heatmap showing the distribution of the nine neuroactives in 156 plant species. The bar plot highlights the sum of the percentage (%) of matches to serotonin/dopamine among the botanical families. c) Percentages of metabolites matched to plantMASST in a human publicly available diet-related dataset (GNPS/MassIVE: MSV000086989)^35,36^ containing fecal samples from vegans (n = 27) and omnivores (n = 27). d) Percentages of metabolites matched to plantMASST in a human diet-related dataset (GNPS/MassIVE: MSV000093005)^37^ containing fecal samples from patients consuming either an American (n = 27) or Mediterranean (n = 82) diet. Boxplots represent first (lower), median, and third (upper) quartiles. Upper and lower whiskers extend to the closest value to +/− 1.5 * interquartile range (IQR). The independent two-sided t-test was applied in Fig 2c, while the two-sided Mann-Whitney-Wilcoxon test was applied in Fig 2d, and statistical significance was observed for p < 0.05. Food icons were obtained from Bioicons.com.

## Data Availability

Data used to generate the reference database of plantMASST are publicly available at GNPS/MassIVE (https://massive.ucsd.edu/). A list with all the accession numbers (MassIVE IDs) of the studies used to generate this tool is available on GitHub (https://github.com/helenamrusso/plantmasst, plant_masst_table.csv). All the taxonomic trees shown in this manuscript can be interactively explored by downloading the .html files available on GitHub (https://github.com/helenamrusso/plantmasst). To help interpret and establish that distinct plant species’ small molecules were only found, known molecules already present in the GNPS library (https://library.gnps2.org/) were employed. - Moroidin (CCMSLIB00005435737) - Piperlongumine (CCMSLIB00010117596) - Caffeine (CCMSLIB00006365672) - Quercetin (CCMSLIB00010118464) - Morin (CCMSLIB00010122829) - Reserpine (CCMSLIB00010110971) - Icaridin (CCMSLIB00000565057) - Lutein (CCMSLIB00005777353) - Methoxsalen (CCMSLIB00006417040) - Cannabidiol (CCMSLIB00009943776) - Tryptophan (CCMSLIB00003136269) - Acetylcholine (CCMSLIB00000578035) - Dopamine (CCMSLIB00006121682) - GABA (CCMSLIB00000215050) - Glutamate (CCMSLIB00000081783) - Norepinephrine (CCMSLIB00000219763) - Serotonin (CCMSLIB00006114036) - THC (CCMSLIB00005774204) - Tryptamine (CCMSLIB00004693658) Data used to search for plant-derived molecules (Figure 2c) from fecal samples of vegans and omnivores is publicly available in GNPS/MassIVE under the accession number MSV000086989. Data used to assess plant-derived molecules in fecal samples from people subjected to an American and Mediterranean diet is publicly available in GNPS/MassIVE under the accession number MSV000093005. Data acquired for retention time matching between piperlongumine standard and plant extracts is available in GNPS/MassIVE under the accession number MSV000094562.

## References

[1] Antonelli A. State of the World’s Plants and Fungi, 2023. https://kew.iro.bl.uk/concern/reports/fccd9838-42a9-401f-a518-e76142164193 (2023) doi:10.34885/WNWN-6S63.

[2] Elser D. Evolutionary metabolomics of specialized metabolism diversification in the genus Nicotiana highlights N-acylnornicotine innovations. Sci Adv 9, eade8984 (2023). 37624884 10.1126/sciadv.ade8984 PMC10456844

[3] Rivière C., Pawlus A. D. & Mérillon J.-M. Natural stilbenoids: distribution in the plant kingdom and chemotaxonomic interest in Vitaceae. Nat. Prod. Rep. 29, 1317–1333 (2012). 23014926 10.1039/c2np20049j

[4] Mannochio-Russo H. Untargeted Metabolomics Sheds Light on the Diversity of Major Classes of Secondary Metabolites in the Malpighiaceae Botanical Family. Front. Plant Sci. 13, 854842 (2022). 35498703 10.3389/fpls.2022.854842 PMC9047359

[5] Kang K. B. Comprehensive mass spectrometry-guided phenotyping of plant specialized metabolites reveals metabolic diversity in the cosmopolitan plant family Rhamnaceae. Plant J. 98, 1134–1144 (2019). 30786088 10.1111/tpj.14292

[6] Sawada Y. RIKEN tandem mass spectral database (ReSpect) for phytochemicals: a plant-specific MS/MS-based data resource and database. Phytochemistry 82, 38–45 (2012). 22867903 10.1016/j.phytochem.2012.07.007

[7] Rutz A. The LOTUS initiative for open knowledge management in natural products research. Elife 11, (2022). 10.7554/eLife.70780 PMC9135406 35616633

[8] Sorokina M., Merseburger P., Rajan K., Yirik M. A. & Steinbeck C. COCONUT online: Collection of Open Natural Products database. J. Cheminform. 13, 2 (2021). 33423696 10.1186/s13321-020-00478-9 PMC7798278

[9] Pilon A. C. NuBBEDB: an updated database to uncover chemical and biological information from Brazilian biodiversity. Sci. Rep. 7, 7215 (2017). 28775335 10.1038/s41598-017-07451-x PMC5543130

[10] NIST Standard Reference Database 1A | NIST. (2014).

[11] MassBank of north America. http://mona.fiehnlab.ucdavis.edu/.

[12] Wang M. Sharing and community curation of mass spectrometry data with Global Natural Products Social Molecular Networking. Nat. Biotechnol. 34, 828–837 (2016). 27504778 10.1038/nbt.3597 PMC5321674

[13] Sorokina M. & Steinbeck C. Review on natural products databases: where to find data in 2020. J. Cheminform. 12, 20 (2020). 33431011 10.1186/s13321-020-00424-9 PMC7118820

[14] Afendi F. M. KNApSAcK family databases: integrated metabolite-plant species databases for multifaceted plant research. Plant Cell Physiol. 53, e1 (2012). 22123792 10.1093/pcp/pcr165

[15] Zuffa S. microbeMASST: a taxonomically informed mass spectrometry search tool for microbial metabolomics data. Nat Microbiol 9, 336–345 (2024). 38316926 10.1038/s41564-023-01575-9 PMC10847041

[16] Jarmusch A. K. ReDU: a framework to find and reanalyze public mass spectrometry data. Nat. Methods 17, 901–904 (2020). 32807955 10.1038/s41592-020-0916-7 PMC7968862

[17] Deutsch E. W. Universal Spectrum Identifier for mass spectra. Nat. Methods 18, 768–770 (2021). 34183830 10.1038/s41592-021-01184-6 PMC8405201

[18] Wang M. Mass spectrometry searches using MASST. Nat. Biotechnol. 38, 23–26 (2020). 31894142 10.1038/s41587-019-0375-9 PMC7236533

[19] Batsoyol N., Pullman B., Wang M., Bandeira N. & Swanson S. P-Massive: A Real-Time Search Engine for a Multi-Terabyte Mass Spectrometry Database. in SC22: International Conference for High Performance Computing, Networking, Storage and Analysis 1–15 (2022).

[20] Schoch C. L. NCBI Taxonomy: a comprehensive update on curation, resources and tools. Database 2020, (2020). 10.1093/database/baaa062 PMC7408187 32761142

[21] Bittremieux W. Universal MS/MS Visualization and Retrieval with the Metabolomics Spectrum Resolver Web Service. bioRxiv 2020.05.09.086066 (2020) doi:10.1101/2020.05.09.086066.

[22] Petras D. GNPS Dashboard: collaborative exploration of mass spectrometry data in the web browser. Nat. Methods 19, 134–136 (2022). 34862502 10.1038/s41592-021-01339-5 PMC8831450

[23] Kersten R. D. Gene-Guided Discovery and Ribosomal Biosynthesis of Moroidin Peptides. J. Am. Chem. Soc. 144, 7686–7692 (2022). 35438481 10.1021/jacs.2c00014

[24] Christina Leung T.-W., Williams D. H., C J Barna J., Foti S. & Oelrichs B., P. Structural studies on the peptide moroidin from laportea moroides. Tetrahedron 42, 3333–3348 (1986).

[25] Morita H., Shimbo K., Shigemori H. & Kobayashi J. Antimitotic activity of moroidin, a bicyclic peptide from the seeds of Celosia argentea. Bioorg. Med. Chem. Lett. 10, 469–471 (2000). 10743950 10.1016/s0960-894x(00)00029-9

[26] Xu X. Moroidin, a Cyclopeptide from the Seeds of Celosia cristata That Induces Apoptosis in A549 Human Lung Cancer Cells. J. Nat. Prod. 85, 1918–1927 (2022). 35951980 10.1021/acs.jnatprod.1c01215

[27] Sumner L. W. Proposed minimum reporting standards for chemical analysis Chemical Analysis Working Group (CAWG) Metabolomics Standards Initiative (MSI). Metabolomics 3, 211–221 (2007). 24039616 10.1007/s11306-007-0082-2 PMC3772505

[28] Zhu P. Overview of piperlongumine analogues and their therapeutic potential. Eur. J. Med. Chem. 220, 113471 (2021). 33930801 10.1016/j.ejmech.2021.113471

[29] Baker C. Allosteric Antagonist Modulation of TRPV2 by Piperlongumine Impairs Glioblastoma Progression. SSRN Electronic Journal Preprint at 10.2139/ssrn.3402071. PMC8161495 34079902

[30] Ramakrishna F. B. S. M. Neurotransmitters in Plant Signaling and Communication. Springer International Publishing).

[31] Tanveer M. & Shabala S. Neurotransmitters in Signalling and Adaptation to Salinity Stress in Plants. in Neurotransmitters in Plant Signaling and Communication (eds. Baluška F., Mukherjee S. & Ramakrishna A.) 49–73 (Springer International Publishing, Cham, 2020).

[32] Dos Santos R. G. & Hallak J. E. C. Ayahuasca, an ancient substance with traditional and contemporary use in neuropsychiatry and neuroscience. Epilepsy Behav. 121, 106300 (2021). 31182391 10.1016/j.yebeh.2019.04.053

[33] Petrucci A. N., Joyal K. G., Purnell B. S. & Buchanan G. F. Serotonin and sudden unexpected death in epilepsy. Exp. Neurol. 325, 113145 (2020). 31866464 10.1016/j.expneurol.2019.113145 PMC7029792

[34] Bagdy G., Kecskemeti V., Riba P. & Jakus R. Serotonin and epilepsy. J. Neurochem. 100, 857–873 (2007). 17212700 10.1111/j.1471-4159.2006.04277.x

[35] NIST Gut Microbiome Metabolomics Interlaboratory Program. https://www.nist.gov/programs-projects/gut-microbiome-metabolomics-interlaboratory-program.

[36] Bayless A. Multi’omic Characterization of Human Whole Stool RGTMs. NIST Internal Report (IR). 10.6028/nist.ir.8451 (2023) doi:10.6028/nist.ir.8451.

[37] Fleming J. A., Kris-Etherton P. M., Petersen K. S. & Baer D. J. Effect of varying quantities of lean beef as part of a Mediterranean-style dietary pattern on lipids and lipoproteins: a randomized crossover controlled feeding trial. Am. J. Clin. Nutr. 113, 1126–1136 (2021). 33826691 10.1093/ajcn/nqaa375 PMC8106750

[38] Chamberlain S. A. & Szöcs E. taxize: taxonomic search and retrieval in R. F1000Res. 2, 191 (2013). 24555091 10.12688/f1000research.2-191.v1 PMC3901538

[39] Huerta-Cepas J., Serra F. & Bork P. ETE 3: Reconstruction, Analysis, and Visualization of Phylogenomic Data. Mol. Biol. Evol. 33, 1635–1638 (2016). 26921390 10.1093/molbev/msw046 PMC4868116

[40] Letunic I. & Bork P. Interactive Tree Of Life (iTOL) v5: an online tool for phylogenetic tree display and annotation. Nucleic Acids Res. 49, W293–W296 (2021). 33885785 10.1093/nar/gkab301 PMC8265157

[41] Allard P.-M. Open and reusable annotated mass spectrometry dataset of a chemodiverse collection of 1,600 plant extracts. Gigascience 12, (2022). 10.1093/gigascience/giac124 PMC9845059 36649739

[42] Magalhães R. Habitual coffee drinkers display a distinct pattern of brain functional connectivity. Mol. Psychiatry 26, 6589–6598 (2021). 33875801 10.1038/s41380-021-01075-4 PMC8760045

[43] Yu X. Metabolite signatures of diverse Camellia sinensis tea populations. Nat. Commun. 11, 5586 (2020). 33149146 10.1038/s41467-020-19441-1 PMC7642434

[44] Li Y. Preclinical reserpine models recapitulating motor and non-motor features of Parkinson’s disease: Roles of epigenetic upregulation of alpha-synuclein and autophagy impairment. Front. Pharmacol. 13, 944376 (2022). 36313295 10.3389/fphar.2022.944376 PMC9597253

[45] Shamon S. D. & Perez M. I. Blood pressure-lowering efficacy of reserpine for primary hypertension. Cochrane Database Syst. Rev. 12, CD007655 (2016). 27997978 10.1002/14651858.CD007655.pub3 PMC6464022

[46] Fradin M. S. 6 - Insect Protection. in Travel Medicine (Fourth Edition) (eds. Keystone J. S. et al.) 43–52 (Elsevier, London, 2019).

[47] Ochoa Becerra M., Mojica Contreras L., Hsieh Lo M., Mateos Díaz J. & Castillo Herrera G. Lutein as a functional food ingredient: Stability and bioavailability. J. Funct. Foods 66, 103771 (2020).

[48] Buscemi S. The Effect of Lutein on Eye and Extra-Eye Health. Nutrients 10, (2018). 10.3390/nu10091321 PMC6164534 30231532

[49] Ham J. R., Choi R.-Y., Lee H.-I. & Lee M.-K. Protective Effects of Methoxsalen Supplementation on Chronic Alcohol-Induced Osteopenia and Steatosis in Rats. Molecules 25, (2020). 10.3390/molecules25051177 PMC7179412 32151025

[50] Ranjan R., Manayath G. J., Salian R. & Narendran V. Methoxsalen-induced advanced bull’s eye maculopathy. Eur. J. Ophthalmol. 31, NP70–NP73 (2021). 32064940 10.1177/1120672120907317

[51] Peng J. A narrative review of molecular mechanism and therapeutic effect of cannabidiol (CBD). Basic Clin. Pharmacol. Toxicol. 130, 439–456 (2022). 35083862 10.1111/bcpt.13710

[52] Arzimanoglou A. Epilepsy and cannabidiol: a guide to treatment. Epileptic Disord. 22, 1–14 (2020). 10.1684/epd.2020.1141 32096470

[53] Legare C. A., Raup-Konsavage W. M. & Vrana K. E. Therapeutic Potential of Cannabis, Cannabidiol, and Cannabinoid-Based Pharmaceuticals. Pharmacology 107, 131–149 (2022). 35093949 10.1159/000521683

[54] Britch S. C., Babalonis S. & Walsh S. L. Cannabidiol: pharmacology and therapeutic targets. Psychopharmacology 238, 9–28 (2021). 33221931 10.1007/s00213-020-05712-8 PMC7796924

