# Supplementary material for: plantMASST - Community-driven chemotaxonomic digitization of plants
Source: bioRxiv. 2024 May 14:2024.05.13.593988. Preprint. [Version 1] doi: 10.1101/2024.05.13.593988 (PMC11118438; doi:10.1101/2024.05.13.593988)
Supplement: Supplement 1 [file NIHPP2024.05.13.593988v1-supplement-1.pdf]

bioRxiv preprint doi: https://doi.org/10.1101/2024.05.13.593988; this version posted May 15, 2024. The copyright holder for this preprint (which was not certified by peer review) is the author/funder, who has granted bioRxiv a license to display the preprint in perpetuity. It is made available under aCC-BY 4.0 International license.

Supplementary information

Complementary use cases

In the plantMASST web interface, a single search was performed using GNPS reference MS/MS spectra of caffeine, and the taxonomic tree was generated as a result (**Supplementary Figure S1b)**. Caffeine is a drug found in many plants; it is a central nervous system stimulant, enhancing wakefulness, improving alertness, and decreasing weariness[42]. The output searches from plantMASST show caffeine was most notably detected in species used to prepare these beverages. For instance, *Camellia sinensis* (Theaceae) is traditionally used for caffeinated teas[43]. Our findings also support caffeine content in cacao (*Theobroma cacao*, Malvaceae), and *Coffea arabica* (Rubiaceae, from which coffee beans are created). Moreover, searching with rare metabolites, such as reserpine was detected in 0.010% of the samples (**Supplementary Figure S3**). Reserpine is a drug that is used clinically to treat illnesses such as hypertension and Parkinson's disease[44]. It was isolated for the first time from the plant *Rauvolfia* (=*Rauwolfia) serpentina* (Apocynaceae) and plays a role by blocking noradrenaline, dopamine, and serotonin reuptake in nerve cells, resulting in relaxing and blood pressure-lowering effects[45]. Reserpine is an example of a natural compound found in plants with medicinal effects, and in this correspondence, given the ability of plantMASST reserpine is being putatively described for the first time in *R. media*, a species from the same genus of *R. serpentina*.

Another utility of plantMASST is for searching for plants that can produce drugs with diverse chemical backbones. Specifically, we examined four approved plant-derived drugs, icaridin, lutein, methoxsalen, and cannabidiol, from the Drugbank database and observed their presence in taxonomically distinct species (**Supplementary Figure S4**). Icaridin is known due to its plant insect-repellent properties. Also, it is often used topically as an insect repellent to protect against mosquito bites, ticks, and other insects[46]. Otherwise, lutein is a naturally occurring carotenoid pigment found in various plants and vegetables, and it is known for its role in eye health[47,48]. It is a major component of the macular pigment in the human retina and is believed to help protect the eyes from oxidative damage and age-related macular degeneration[48]. Methoxsalen is a naturally occurring compound found in certain plants, including citrus fruits like oranges and lemons, as well as in various plants such as figs and parsley[49]. Methoxsalen is primarily used in a medical procedure known as PUVA (psoralen plus ultraviolet A) therapy, and it is also used to treat skin conditions like psoriasis, vitiligo, and eczema[50].

bioRxiv preprint doi: https://doi.org/10.1101/2024.05.13.593988; this version posted May 15, 2024. The copyright holder for this preprint (which was not certified by peer review) is the author/funder, who has granted bioRxiv a license to display the preprint in perpetuity. It is made available under aCC-BY 4.0 International license.

Last, cannabidiol (CBD) is one of the many compounds derived from the resin of cannabis flowers and can be extracted for various uses[51]. CBD has gained significant attention for its potential medicinal benefits. It is used in the treatment of epilepsy (specifically, Dravet syndrome and Lennox-Gastaut syndrome), chronic pain management, anxiety, and as an anti-inflammatory agent[52–54]. Thus, plantMASST allowed us to find these drugs in different families *e.g.*, icaridin was detected in species from several families. Otherwise, the other drugs were mainly detected in specific species, for instance, lutein in *Eremophila calcicola* (Scrophulariaceae), methoxsalen in *Citrus* (Rutaceae), and CBD in *Cannabis sativa* (Cannabaceae). Therefore, these findings indicated that the plantMASST can be leveraged to hypothesize the discovery of abundant sources of plants containing structurally analogous drugs and their potential origins of these drugs/MS-MS signals from a database containing metabolome information from extracts of plants. In other cases, such as tryptophan, it is very frequently detected (18.69% of all the samples, **Supplementary Figure S6**), highlighting that plantMASST can be leveraged to hypothesize if the MS/MS is a taxonomically common or rare metabolite.

bioRxiv preprint doi: https://doi.org/10.1101/2024.05.13.593988; this version posted May 15, 2024. The copyright holder for this preprint (which was not certified by peer review) is the author/funder, who has granted bioRxiv a license to display the preprint in perpetuity. It is made available under aCC-BY 4.0 International license.

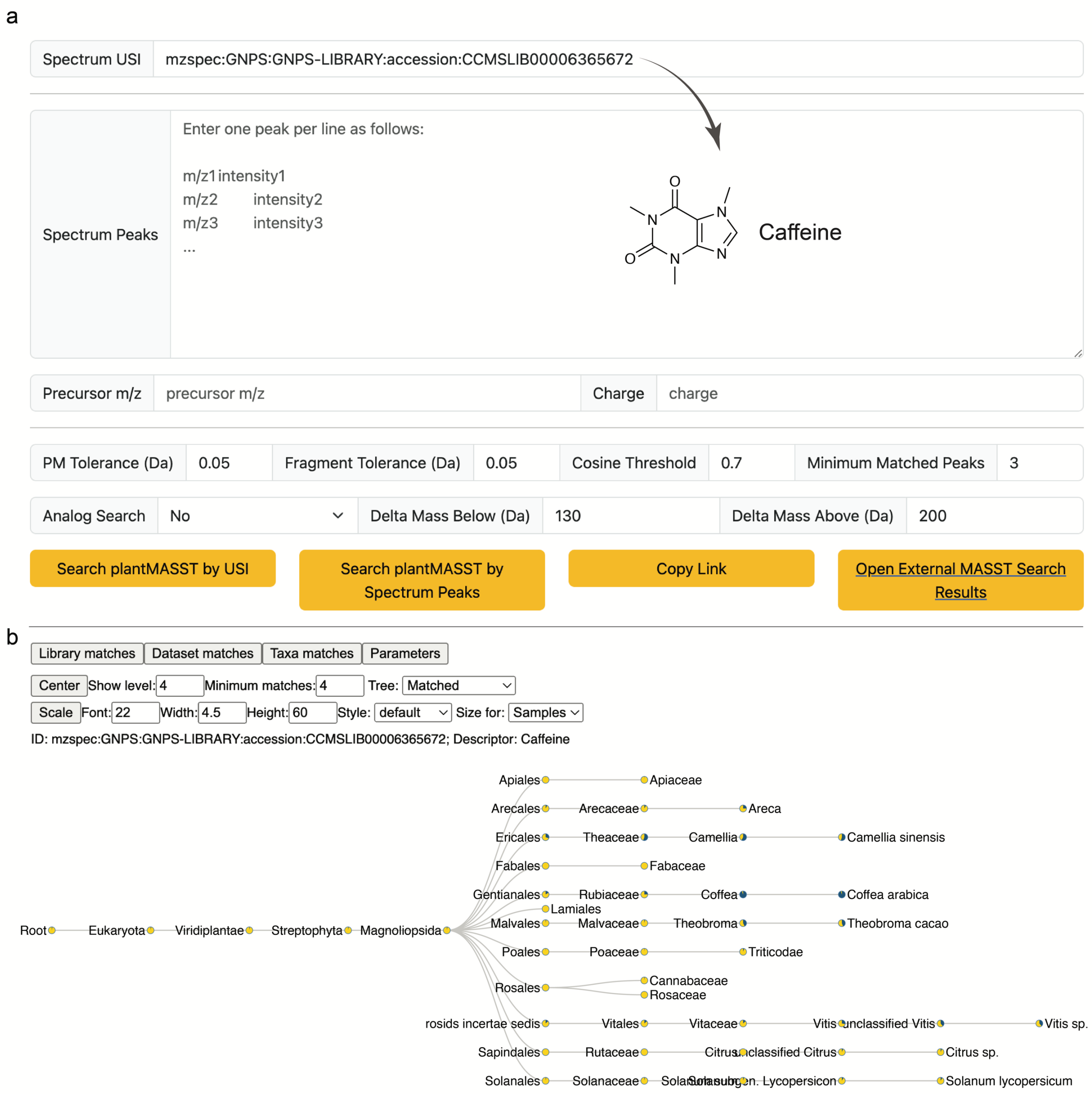

Supplementary Figure S1. plantMASST web app. a) The plantMASST web app is available at https://masst.gnps2.org/plantmasst/. People can search the plantMASST reference database for MS/MS spectra by entering a USI in 'Spectrum USI' or fragment ions, intensities, and precursor mass in 'Spectrum Peak' and 'Precursor *m/z*', respectively. Precursor and fragment ion tolerances, cosine threshold, and minimum matching peaks are adjustable. Analog search is also available. Finally, based on the information presented, users can enter a search query by clicking either 'Search plantMASST by USI' or 'Search plantMASST by Spectrum Peak'. Search jobs can be readily shared by clicking on the 'Copy Link' button. Reference MS/MS spectrum (CCMSLIB00006365672) available in the GNPS library was used to search for caffeine in the plantMASST database. b) interactive taxonomic tree and distribution of caffeine in plant species according to the NCBI taxonomy. Also, links to related tools in the GNPS ecosystem are provided (library, dataset, and taxa matches). These matches represent level 2 annotations according to the Metabolomics Standards Initiative[27].

bioRxiv preprint doi: https://doi.org/10.1101/2024.05.13.593988; this version posted May 15, 2024. The copyright holder for this preprint (which was not certified by peer review) is the author/funder, who has granted bioRxiv a license to display the preprint in perpetuity. It is made available under aCC-BY 4.0 International license.

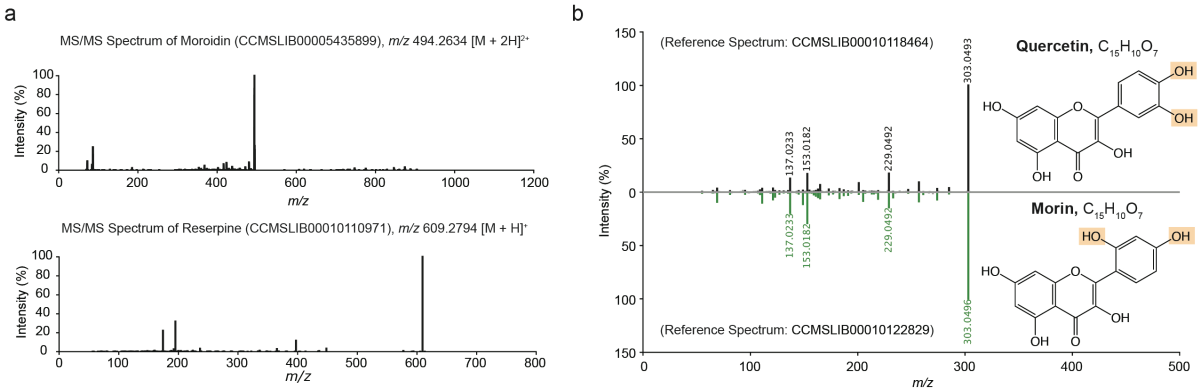

Supplementary Figure S2. High-resolution MS/MS spectra of moroidin and reserpine. a) Single spectrum of moroidin and reserpine. b) Mirror plot between the MS/MS spectra of quercetin and morin. Although they have the same spectrum, their structures are different.

bioRxiv preprint doi: https://doi.org/10.1101/2024.05.13.593988; this version posted May 15, 2024. The copyright holder for this preprint (which was not certified by peer review) is the author/funder, who has granted bioRxiv a license to display the preprint in perpetuity. It is made available under aCC-BY 4.0 International license.

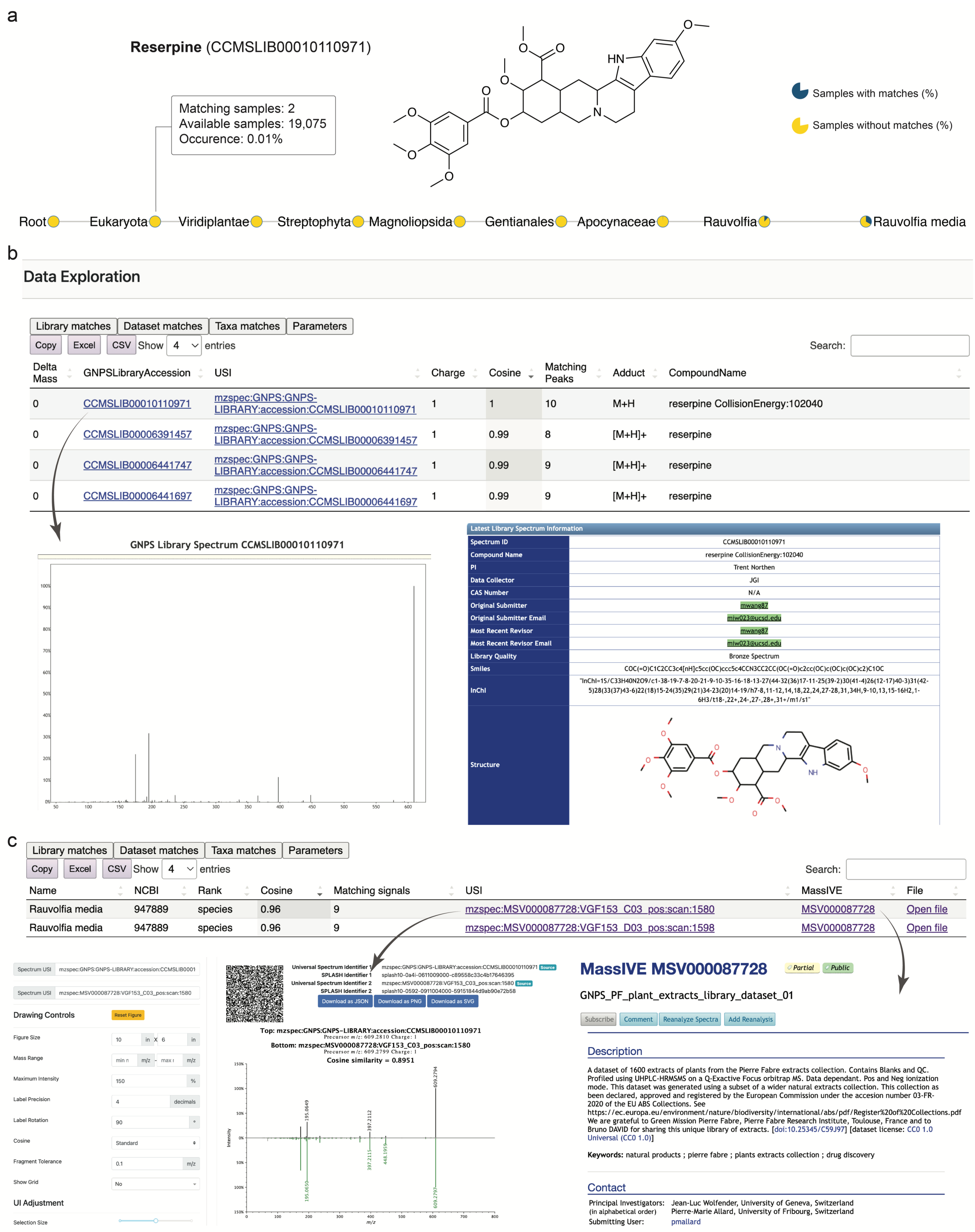

Supplementary Figure S3. Complementary output of plantMASST. a) Example of plantMASST taxonomic tree of reserpine, known to be produced by specific plants. The taxonomic tree shows the MS/MS spectrum of reserpine searched against the plantMASST database was detected only in *Ochrosia elliptica.* It represents 0.010% of the samples available in the database (2 samples out of 19,075). Pie charts display

bioRxiv preprint doi: https://doi.org/10.1101/2024.05.13.593988; this version posted May 15, 2024. The copyright holder for this preprint (which was not certified by peer review) is the author/funder, who has granted bioRxiv a license to display the preprint in perpetuity. It is made available under aCC-BY 4.0 International license.

the percentage of matches within that taxonomic level detected against the plantMASST database. Blue indicates the percentage of samples with matches and yellow without matches. Reference MS/MS spectra of reserpine (CCMSLIB00010110971), and it represents a level 2 annotation according to the Metabolomics Standards Initiative[27]. b) The MS/MS spectrum is searched against the GNPS libraries, and possible annotations are returned if matches are identified. Users can visit the accompanying GNPS Library Spectrum page for information on the reference spectrum. c) Data on matched scans in the sample from various taxa is supplied. Furthermore, users can visualize the mirror plot between the queried spectrum and spectrum from datasets included in the plantMASST database, such as the similarity score, and matching fragments. The user can also obtain the project's MassIVE accession number as well as contact information for the person who contributed the data.

bioRxiv preprint doi: https://doi.org/10.1101/2024.05.13.593988; this version posted May 15, 2024. The copyright holder for this preprint (which was not certified by peer review) is the author/funder, who has granted bioRxiv a license to display the preprint in perpetuity. It is made available under aCC-BY 4.0 International license.

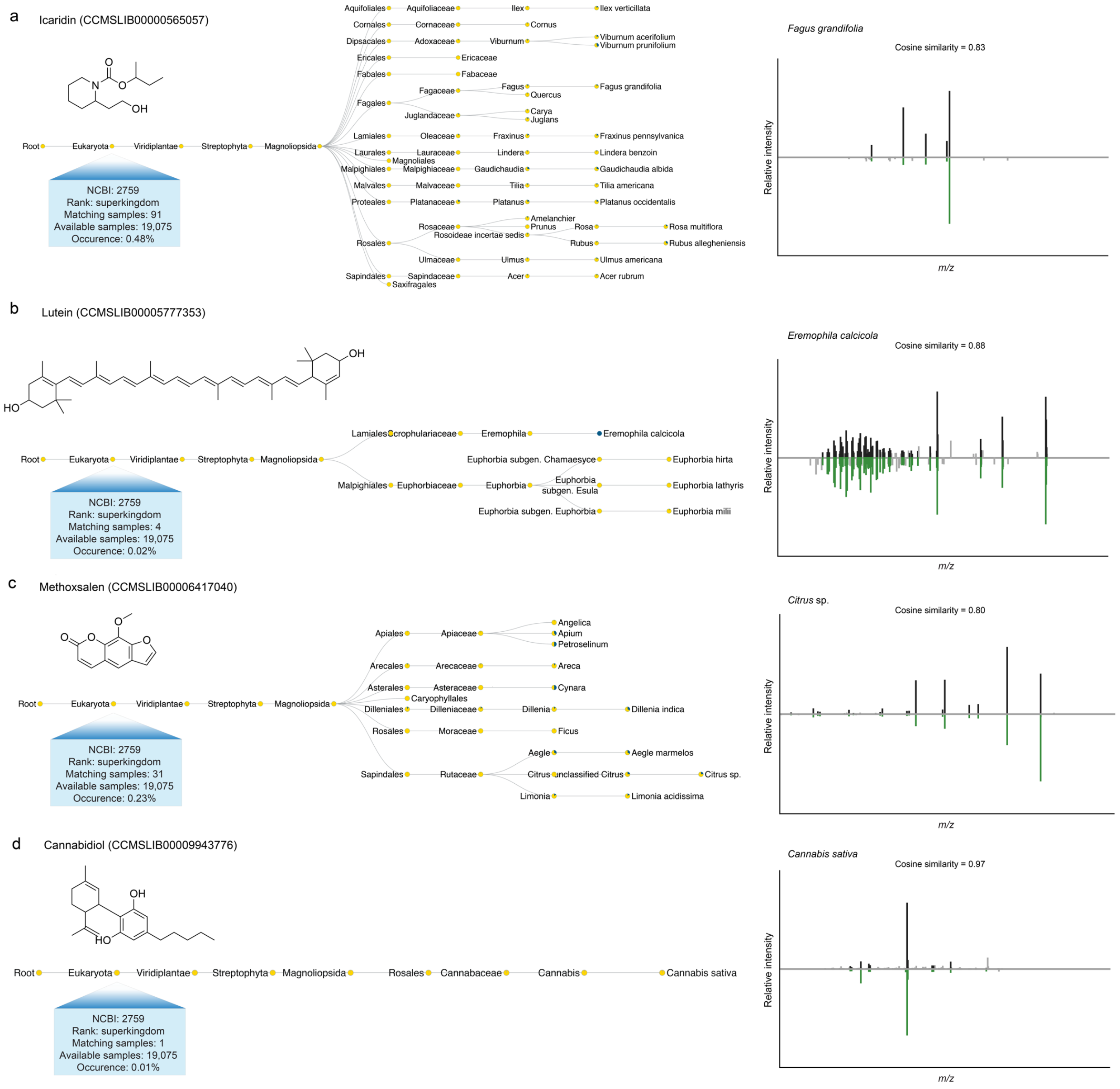

Supplementary Figure S4. plantMASST examples of four rarely occurring plant-derived drugs. The plantMASST taxonomic trees for four drugs: a) icaridin, b) lutein, c) methoxsalen, and d) cannabidiol. Outputs showcase their occurrences in the reference datasets of plantMASST. The pie charts represent the percentage of matches within each taxonomic level detected against the plantMASST database. Blue indicates the percentage of samples with matches, while yellow without matches. The mirror plots show the matches between the representative MS/MS spectrum (top) of each drug and their closely matched MS/MS spectrum (bottom) in the plantMASST database. These are level 2 annotations according to the Metabolomics Standards Initiative[27]. a) icaridin, a piperidine-type alkaloid used as an insect repellent, was observed in Malpighiaceae plants known for their insecticidal and repellent properties. b) lutein, a carotenoid, was detected in various plant families, including Euphorbiaceae, which is a notable source of lutein. c) methoxsalen, a furanocoumarin known for causing photosensitization of the skin, was found in alternative plant sources with no prior references related to methoxsalen. d) cannabidiol, a cannabinoid used for various medical purposes, was observed in *Cannabis sativa*, known as the sole source of

bioRxiv preprint doi: https://doi.org/10.1101/2024.05.13.593988; this version posted May 15, 2024. The copyright holder for this preprint (which was not certified by peer review) is the author/funder, who has granted bioRxiv a license to display the preprint in perpetuity. It is made available under aCC-BY 4.0 International license.

cannabidiol.

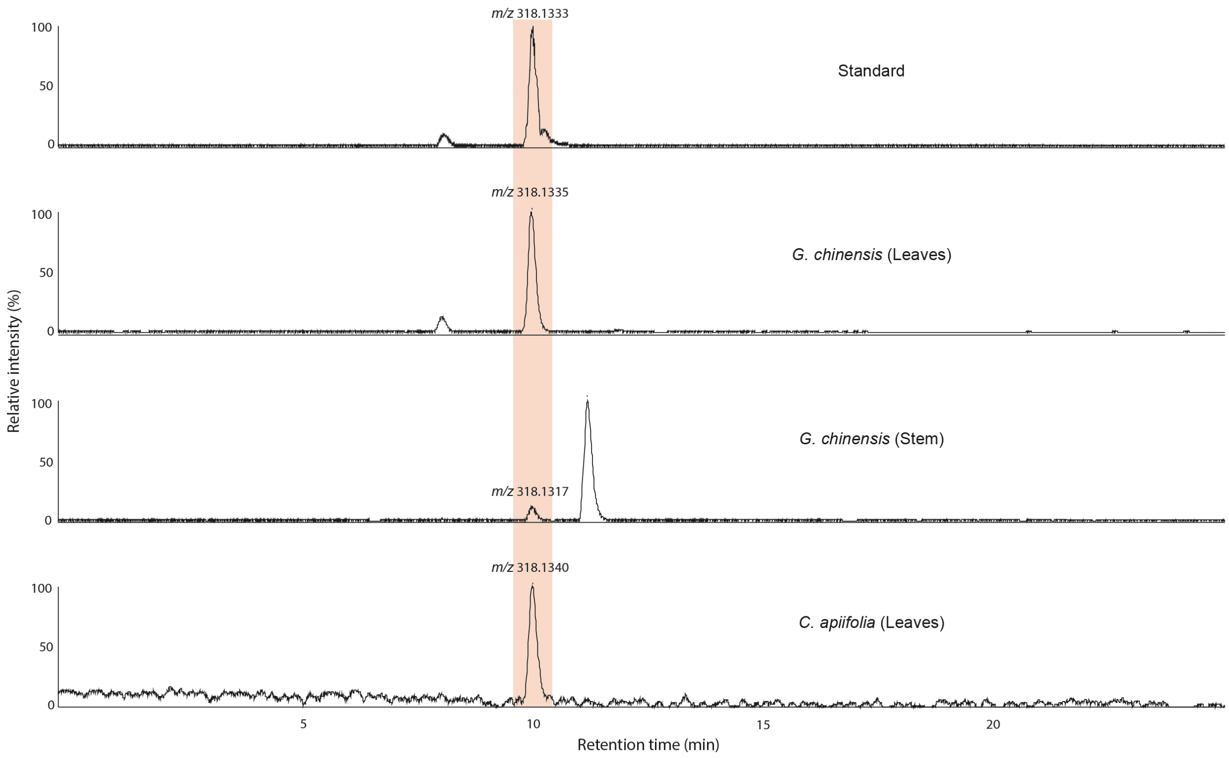

Supplementary Figure S5. Retention time matching with piperlongumine. Retention time matching between piperlongumine (*m/z* 318, $[M + H]^+$) commercial standards and *Gymnotheca chinensis* (leaves and stem) and *Clematis apiifolia* (leaves) extracts. Piperlongumine was not observed in the other extracts analyzed (*C. apiifolia* stems and roots). These are level 1 identifications according to the Metabolomics Standards Initiative[27].

bioRxiv preprint doi: https://doi.org/10.1101/2024.05.13.593988; this version posted May 15, 2024. The copyright holder for this preprint (which was not certified by peer review) is the author/funder, who has granted bioRxiv a license to display the preprint in perpetuity. It is made available under aCC-BY 4.0 International license.

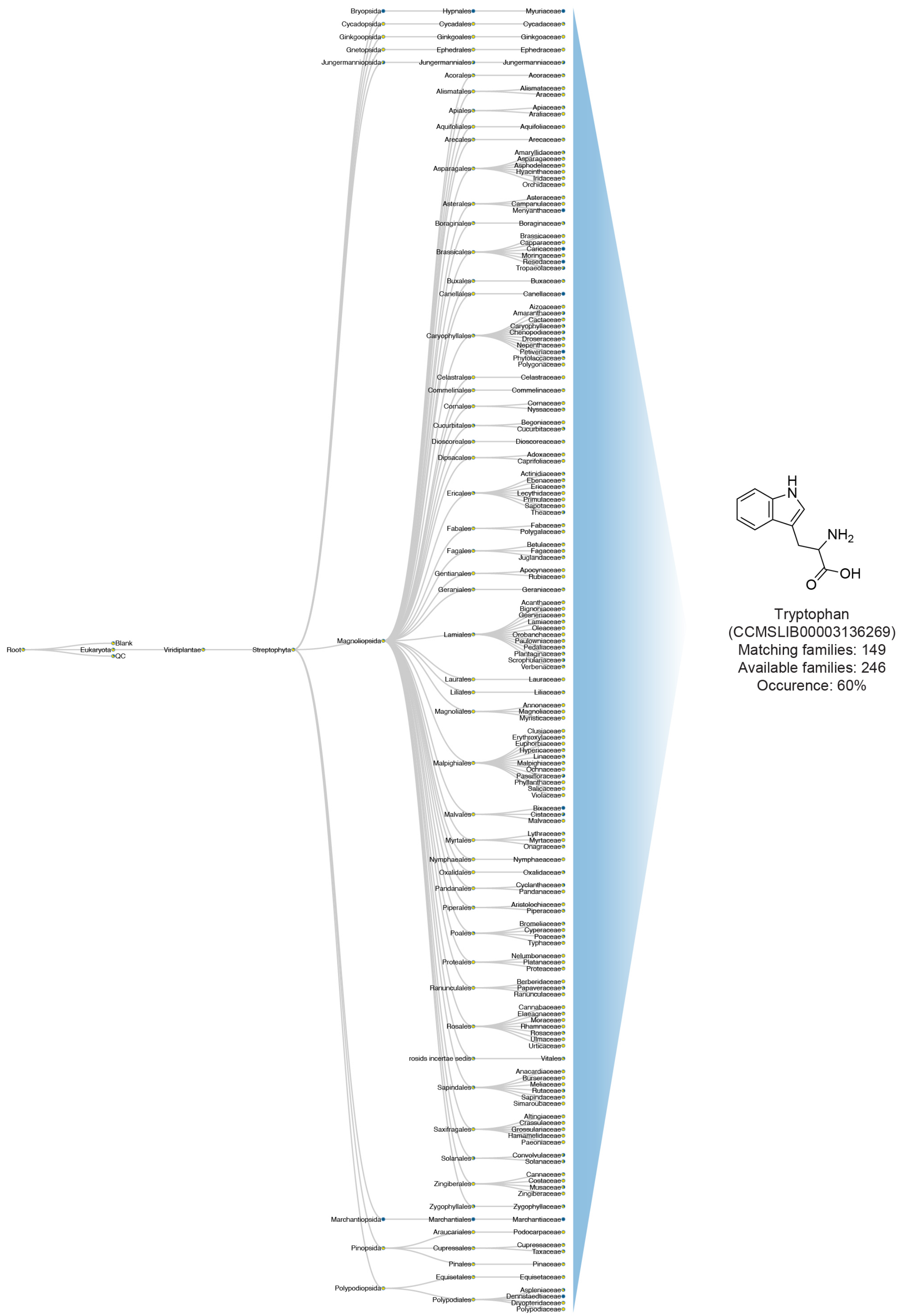

Supplementary Figure S6. Taxonomic tree of tryptophan. Taxonomic tree of tryptophan (CCMSLIB00003136269) and its distribution abroad plant families according to the NCBI taxonomy.
